# Attitudes toward COVID-19 vaccination of healthcare workers in Israel and vaccination rates during vaccine rollout

**DOI:** 10.1017/S0950268823000961

**Published:** 2023-07-24

**Authors:** Assaf Halevi, Vered Shkalim Zemer, Tami Embar, Eyal Jacobson, Yael Reichenberg, Noga Yosef, Dekel Shlomi

**Affiliations:** 1 Adelson School of Medicine, Ariel University, Ariel, Israel; 2 Sackler Faculty of Medicine, Tel Aviv University, Tel Aviv, Israel; 3 Em Hamoshavot clinic, Clalit Health Services Community Division, Petah-Tiqwa, Israel; 4 Research and Assessment Department, Clalit Health Service, Tel-Aviv, Israel; 5 Clalit Supplementary Health Services, Clalit Health Service, Bnei-Brak, Israel; 6 Managment, Dan- Petah-Tiqwa District, Clalit Health Services Community Division, Ramat-Gan, Israel; 7 Research Unit, Dan- Petah-Tiqwa District, Clalit Health Services Community Division, Ramat-Gan, Israel; 8 Pulmonary Clinic, Dan- Petah-Tiqwa District, Clalit Health Services Community Division, Ramat-Gan, Israel

**Keywords:** COVID-19, healthcare workers, survey, vaccine acceptance, vaccine hesitancy

## Abstract

A new COVID-19 vaccine was introduced in a remarkably short period of time. Public and healthcare workers (HCWs) were concerned about the safety of the vaccine, especially in light of the use of new technologies. A review regarding attitudes towards COVID-19 vaccination found a 22.5% hesitancy rate among HCWs. Online anonymous questionnaires were delivered using a web-based surveying platform to community HCWs in a central district in Israel from 3 to 19 January 2021. The real COVID-19 vaccination data were collected between the beginning of the vaccination rollout and the end of the month after the survey as well as the real vaccination rate among the general population. Of the 3,172 HCWs, 549 (17%) responded to the questionnaire. The highest positive attitude towards the vaccine was among physicians (95%), while nurses showed the highest level of hesitation (14%) for a specific sector (*P* < 0.05). However, the real vaccination rates were similar among physicians (63%) and nurses (62%). Surprisingly, the total vaccination rate of HCWs was substantially lower (52%) than that of the general population (71%). The main vaccination motivators were the social and economic effects of the COVID-19 epidemic. Focused strategies to reduce the level of hesitancy among HCWs are needed.

## Introduction

In March 2020, COVID-19 (caused by severe acute respiratory syndrome coronavirus 2 (SARS-CoV-2)) was recognised by the World Health Organization (WHO) as a worldwide pandemic. It went on to affect over 5 million people across 215 countries or territories, causing more than 300,000 fatalities worldwide by the end of May 2020 [1, 2].

The first vaccine to be authorised was the Pfizer–BioNTech COVID-19 vaccine (BioNTech (BNT) 162b2 mRNA), which was approved by the FDA with Emergency Use Authorisation (EUA) on 11 December 2020 [1]. In Israel, the administration of the vaccine began on 19 December 2020, in an official government operation called ‘Give a Shoulder’ [2]. At that time, Israel experienced a second wave of morbidity, with 2,734 new cases on 19 December (294.26 per million) and 3,074 cumulative deaths (330.86 per million) [3].

For vaccines to be effective in a pandemic, they should provide ‘herd immunity’ by exceeding a threshold rate of vaccinating many people. However, although the scientific and medical community demonstrated a remarkable and one-of-a-kind success in developing fast and very efficient vaccines, these fast and new technologies translated into fears in many sectors of society, including among physicians and nurses. Strategies to increase adherence to vaccination and to identify the contributing factors affecting vaccine acceptance were studied in many countries. Healthcare workers (HCWs) played a key role in influencing the general public’s decisions about whether or not to receive the COVID-19 vaccine [4, 5].

In a systematic review conducted in January 2021 which studied the attitudes towards vaccination uptake for COVID-19, it was found that the lowest rates (below 60%) were in Italy, Russia, Poland, the United States, France, Kuwait, and Jordan, whilst the highest rates (above 90%) were in Ecuador, Malaysia, Indonesia, and China [7].

In studies from the general population, it has been shown that COVID-19 vaccination hesitancy, and vaccine hesitancy in general, is a complex decision-making process that involves cultural, sociodemographic, emotional, and political factors [4, 6]. Higher odds of vaccine hesitancy were found in persons with lower levels of concerns about being infected as compared to very concerned ((Odds ratio (OR) = 3.80; 95% confidence interval (CI) 2.39–6.03, *P* = 0.001)), and with a lower perception level of the likelihood of being infected (definitely not versus very likely, OR = 6.47; 95% CI 3.74–11.21, *P* < 0.001) [7].

A few studies conducted in late 2020 and early 2021 suggest that many HCWs also have doubts regarding vaccinations, with the highest hesitancy rate measured in Congo and the lowest in China (72% and 4.3%, respectively). In the USA, the hesitancy rates in different countries range between 8% and 18% [8–11]. A review of 35 studies by Biswas et al. was performed to explore the nature and extent of COVID‑19 vaccination hesitancy among HCWs in different countries and their main concerns [8]. The average hesitancy rate of HCWs was 22.5%, with very similar concerns to those identified in the general population. One of those studies was conducted in Israel in March 2020, in which 1,941 anonymous questionnaires were completed by hospital and community HCWs and members of the general population regarding the positive attitude towards a potential COVID-19 vaccine [11]. This study revealed that vaccine-positive attitudes among nurses were significantly lower than those among physicians and the general population (61%, 78%, 75%, respectively, *P* < 0.01). HCWs in internal medicine departments displayed a significantly higher positive attitude than those in general surgery departments (91% versus 75%, respectively, *P* < 0.01).

The aim of this study is to explore attitudes towards the new COVID-19 vaccine among community HCWs in the centre of Israel at the beginning of the mass vaccination drives. Additionally, we explored the factors that affect their intention to accept the vaccine and the reasons for hesitancy or refusal to accept the vaccine. Then, we compared the questionnaire results to the real documented vaccination rate of the HCWs and the general population in the district during the same period.

## Materials and methods

This cross-sectional study was conducted in the Dan-Petah-Tiqwa district (in the centre of Israel) of ‘Clalit Health Services’ (CHS), the largest publicly funded Health Maintenance Organization (HMO) in Israel. The study population consisted of all HCWs aged ≥18 years in the district. Online anonymous questionnaires were delivered between 3 January and 19 January 2021, using Google Forms as a surveying platform. Using ‘Nemala+’ platform, Top Solutions (L.H.B) Ltd. [12], we calculated the sample size (minimal response rate) needed in our population of 3,172 health workers to be 506 for a 95% CI and a 4% margin of error. The questionnaire included sociodemographic data (age group, gender, and healthcare sector) and attitudes towards COVID-19 vaccination questions (intention to be vaccinated and the reason for willingness or avoidance). Participants were also asked to grade their tendency to try new experiences such as new food. Data regarding the real COVID-19 vaccination among these HCWs between the beginning of vaccination (19 December 2020) and the end of the next month after the survey (28 February 2021) were also extracted and analysed. Participants were divided into five sector groups: physicians, nurses, administration & logistics, pharmacists, and other health professions (physiotherapy, occupational therapy, dietitian, speech-therapy, social work, psychology, paramedics, medics, phlebotomist, and medical technicians). The study was approved by the institutional review board (IRB) of Meir Medical Center, Kfar-Saba, Israel (approval no. COM1–0087-21). Since the survey was conducted anonymously and all other data were retrieved retrospectively, the IRB approved conducting this study without obtaining signed informed consent from the study participants.

### Statistical analysis

Data were analysed using IBM SPSS Statistics version 21 (IBM, Armonk, NY). Chi-square and Cramer’s tests were used to compare categorical data. The distribution of the data was evaluated with the Kolmogorov–Smirnov test. The Mann–Whitney U test and Kruskal–Wallis test were used.

## Results

During 17 days in January 2021, 549 out of 3,172 (17%) HCWs in the Dan-Petah-Tiqwa district answered the questionnaires. The demographic and general characteristics of the participants are described in Table 1. Most of the participants – 414 (75.4%) – were female, but no gender difference was found with respect to vaccination attitude. The majority of the HCWs who answered the questionnaires were in the 31–50 age group (279, 51%). The vaccine-positive attitude rates were higher as age increased (92% at the age of ≥51 versus 67% between 18 and 30 years, *P* = 0.001).

**Table 1.** Survey participant characteristics

	Vaccination Attitudes				
Characteristic	Total answers	Vaccinated or intend to be vaccinated	Hesitate, wait or avoid	Irrelevant	*p*-value
Age group					0.001
18-30	43 (8%)	29 (67%)	9 (21%)	5 (12%)	
31-50	279 (51%)	243 (87%)	25 (9%)	11 (4%)	
51+	227 (41%)	208 (92%)	12 (5%)	7 (3%)	
Total	549	480 (88%)	46 (8%)	23 (4%)	
Gender					0.193
Male	126 (23%)	114 (90%)	7 (6%)	5 (4%)	
Female	414 (77%)	358 (86%)	38 (9%)	18 (4%)	
Total	540	472 (88%)	45 (8%)	23 (4%)	
Sector					0.05
Physicians	149 (27%)	141 (95%)	6 (4%)	2 (1%)	
Nurses	139 (25%)	114 (82%)	19 (14%)	6 (4%)	
Pharmacists	35 (6%)	31 (89%)	2 (6%)	2 (6%)	
Administration & Logistics	123 (22%)	107 (87%)	10 (8%)	6 (5%)	
Other health professions^a^	102 (19%)	86 (84%)	9 (9%)	7 (7%)	
Total	548	479 (88%)	46 (8%)	23 (4%)	

a Health professions consist of physiotherapy, occupational therapy, dietitian, speech-therapy, social work, psychology, paramedics, medics, phlebotomist, and medical technicians.

The highest positive attitude towards the vaccine was among physicians (95%), while nurses showed the highest level of hesitation (14%) for a specific sector (*P* = 0.05). The real documented vaccination rates of the HCWs in the Dan-Petah-Tiqwa district between the beginning of the vaccination drive and the end of the month after the survey show a different picture (Table 2). The average vaccination rate was 52%. The highest vaccination rates were similar among physicians (63%) and nurses (62%), while the lowest vaccination rates were among the administration and logistics (41%) and other health profession (45%) sectors (*P* < 0.001). Unfortunately, data regarding the gender and age of HCWs who were vaccinated were inaccessible due to the privacy policy.

**Table 2.** Documented vaccination data of HCWs

Sector	Vaccination status			
	Total	Vaccinated (%)	Not vaccinated (%)	*p*-value
Physicians	748 (23%)	474 (63%)	274 (37%)	< 0.001
Nurses	410 (13%)	253 (62%)	157 (38%)	
Pharmacists	219 (7%)	122 (56%)	97 (44%)	
Administration & Logistics	1130 (36%)	464 (41%)	666 (59%)	
Other health professions^a^	665 (21%)	332 (50%)	333 (50%)	
Total	3,172	1,645 (52%)	1,527 (48%)	

a Health professions consist of physiotherapy, occupational therapy, dietitian, speech-therapy, social work, psychology, paramedics, medics, phlebotomist, and medical technicians.

The data regarding the general population vaccination rate in the district are described in Table 3. The age of retirement in Israel is 67, yet many continue to work for a few more years; hence, the data refer to ages 18–70 to match the ages of the respondents to the survey. As expected, the vaccination rates increased as a function of age (*P* < 0.001). No significant differences were found between genders.

**Table 3.** Vaccination data of the general population in the district

Characteristic	Vaccination status			*p*-value
	Total (%)	Vaccinated (%)	Not vaccinated (%)	
Age group				< 0.001
18-30	68,778 (25%)	38,795 (56%)	29,983 (44%)	
31-50	124,373 (45%)	89,369 (72%)	35,004 (28%)	
51-70	81,448 (30%)	67,616 (83%)	13,832 (17%)	
Gender				0.086
Male	133,780 (49%)	95,584 (71%)	38,196 (29%)	
Female	140,819 (51%)	100,196 (71%)	40,623 (29%)	
Total	2,74,599	195,780 (71%)	78,819 (29%)	

Most of the study participants (397, 72.3%) declared that they were already vaccinated with the first dose, and 15.1% declared that they were willing to be vaccinated. A small group stated that they would postpone vaccination until a mass of people were vaccinated (4.4%). Only a small percentage declared that they were hesitant or not intending to receive the vaccine (1.6% and 1.8%, respectively).

In the survey, participants mentioned reasons for accepting or declining the vaccine (Table 4). The major reasons for vaccine acceptance were personal (fear of being infected or infecting family members with COVID-19) and social (decreasing overall morbidity and improving the economic state). The two main reasons for hesitancy were fear of the rapid development process of the COVID-19 vaccine (52.5%) and the unknown side effects of the COVID-19 vaccine in Israel (50%). Among the survey participants, 10% believed that the COVID-19 vaccine was unnecessary, and 2.5% believed that any kind of vaccine was unnecessary.

**Table 4.** Reasons for COVID-19 vaccine acceptance or hesitancy

	Participants who replied No. (%)	Answers (%)
Reasons for positive attitude towards vaccination*		
I wish the morbidity rate will decrease and economic situation will recover	285 (57.2)	26.4
I am afraid to infect my family members with COVID-19	281 (56.4)	26
I am afraid to be infected by COVID-19	258 (51.8)	23.9
I wish that the COVID-19 limitaions on transportation in the country or on travelling abroad will ease	127 (25.5)	11.7
Reasons for vaccine hesitancy or refusal^a^		
I am afraid of the rapid development of the COVID-19 vaccine	21 (52.5)	31
I am interested in knowing the side effects of the COVID-19 vaccine in Israel	20 (50)	30
I am interested in the COVID-19 vaccine of another company	6 (15)	9
I am interested in other COVID-19 vaccine technology	6 (15)	9
I think the COVID-19 vaccine is unnecessary in general	4 (10)	6
I think vaccines of any kind are unnecessary	1 (2.5)	1.5
other^b^	9 (22.5)	13

a Respondents were able to choose more than one answer.

b Other reasons for COVID-19 vaccine hesitancy or refusal included: There is scarce information about COVID-9 vaccine in pregnant women; Pregnancy; My husband and children were ill with COVID-19 while I had a negative PCR-COVID-19 test but I’m waiting to perform a COVID-19 serology test; Women at fertility period or having fertility treatments; I follow the instructions for wearing a mask, domestic isolation and exit limitations to public space and do not want to expose myself to chemicals; I heard the COVID-19 vaccine was not tested on women at fertility age and therefore I am afraid to get vaccinated; I heard that the COVID-19 vaccine may impair fertility; ; I am at the beginning of my pregnancy and the COVID-19 vaccine was not tested on women in my condition.

An additional question referred to the tendency to experience and enjoy new behaviours. Forty-one percent of the vaccinated and 48% of the hesitators replied that they enjoyed it very much. Additionally, 21% and 30% of the vaccinated and hesitator groups, respectively, answered that they enjoy new behaviours to a small extent or not at all. No correlation was found between the answers to this question and attitudes towards the COVID-19 vaccine.

## Discussion

This study examined the attitudes of HCWs in one district of CHS towards the COVID-19 vaccine at the beginning of the vaccination rollout in Israel through a survey. Survey answers were compared to the real vaccination status of the HCWs as well as the general population from the beginning of the vaccination rollout to the end of the month after the survey. Out of 3,172 HCWs in our district, 549 (17%) responded to the survey. Our study met the minimal criteria for the calculated response rate of 506 (16%) completed surveys. According to the survey results, the most prominent factor for holding a positive attitude towards the COVID-19 vaccine is age (67% versus 92% among 18–30 and ≥ 51 years old, respectively, *P* = 0.001), while gender has no influence on vaccine-positive attitudes (Table 1).

According to the survey, an MD degree expressed the highest pro-vaccine attitude, while nurses had the lowest positive attitude towards vaccination (95% versus 82%, respectively, *P* = 0.05). Other surveys showed similar results in which nurses expressed a higher hesitancy rate towards the COVID-19 vaccine. For example, as mentioned in the introduction, in Israel at the beginning of the pandemic, nurses had lower positive attitudes than physicians and the general population (61%, 78%, 75%, respectively, *P* < 0.01) [11]. A study conducted in Greece in 2020 among 500 HCWs in five hospitals found higher positive attitudes among physicians (60.7%) than among nurses (34%) and paramedics (43.5%, *P* < 0.001) [13]. A survey among 1,398 HCWs in the USA found higher rates of positive attitudes towards COVID-19 vaccination among physicians than among nurses (94.5% versus 77.7%, respectively, difference = 16.8%, 95%; CI 9.5–24.2) [14]. Another study was conducted among 12,034 HCWs in two hospitals in Philadelphia in December 2020 [15]. Among 11 variables that were significantly associated with intention or hesitation to receive the vaccine, the two most influential factors were age ( ≥ 65 versus < 40, OR = 3.50; 95% CI 2.50–4.90, *P* < 0.0001) and the level of education (postgraduate degree versus less than a Bachelor’s degree, OR = 4.59; 95% CI 3.83–5.50, *P* < 0.0001). Unlike our finding, another significant factor in this study was that the male gender had a higher likelihood of having a positive attitude towards a future vaccine than the females (OR = 2.41, 95% CI 2.12–2.75, *P* < 0.001), presumably due to increased morbidity and mortality associated with COVID-19 in males. The male gender was also found to be an enabling factor for vaccination in 25 of 35 (71%) of the studies in a comprehensive review of attitudes towards the COVID-19 vaccine, in addition to older age and a higher level of education (23 of 35 studies) [8].

In the real documented vaccination data of the HCWs, at the end of the month after the survey, the total vaccination rate was substantially lower (52%) than the positive attitudes, as was demonstrated in the survey (88%). There were no differences between physicians and nurses (63% and 62%, respectively), while administration and logistics workers demonstrated the highest difference between the intention to be vaccinated (87%) and the real vaccination data (41%). It appears that more physicians with a positive attitude towards the vaccine (95%) were more willing to answer the questionnaire than physicians in other sectors. However, in real life, the overall vaccination rate was lower and with less difference between physicians and nurses. Another possible explanation is social desirability bias, which is the tendency of respondents to answer in a way they believe will be considered positively by the reviewers [16]. Physicians are more expected to show a positive attitude towards the vaccine coupled with the time in which the survey was conducted in which a major government campaign calling for mass vaccination was initiated. The lowest vaccination rate among the administration and logistics workers could be explained by the high proportion of young people in these sectors (‘National Service’ duty as a replacement for mandatory military service).

A surprising fact was that in the real vaccination data (at least one vaccine), the general population in the district had a higher rate (71%) than the HCWs (52%) at the same time. However, age and gender analysis could not be performed for the HCWs, as mentioned above. Those high rates of vaccination in a noticeably short duration are globally unique. According to the global database of COVID-19 vaccinations, on 28 February 2021, 50.92% of the Israeli population received at least one vaccine dose, compared to the United Kingdom, Bahrain, and the United States, which have the closest rates (41.7%, 20.77%, and 15.9%, respectively) [3].

As expected, age in the general population had a significant contribution to vaccine acceptance (*P* < 0.001). The age finding is in line with the known literature. A scoping review summarising the key findings of 22 studies conducted in August 2021 counted age group among the most common factors influencing attitudes towards the COVID-19 vaccine along with gender education and employment status [17]. One example is from a global study conducted in June 2020, a survey of 13,462 participants from 19 countries that found that age was a significant factor for a positive attitude towards COVID-19 vaccination, with the highest positive attitude among older individuals (65+) compared to younger individuals (18–24, OR = 1.73; 95% CI 1.48–2.02) [18].

Gender in our study had no effect on actual vaccination in the general population; however, in two studies from the UK (n = 2,025) and Ireland (n = 1041), females were significantly more hesitant (OR = 1.43 and 1.62 in the UK and Ireland, respectively) [19]. Another study conducted in the US at the beginning of vaccination also found that men are overall less likely than women to have vaccine hesitancy (log OR, −0.13; *P* < 0.001) [20].

The average hesitancy rate in our survey was 8% (Table 1), which is relatively low compared to the average hesitancy rate of HCWs (22.5%) that was found in Biswas et al.’s review [8]. Among them, another study in a medical centre in northern Israel that was conducted a year before (in March 2020) found a hesitancy rate of 28.5% among HCWs [11]. This difference may be partially explained by the fact that the former Israeli study was conducted 7 months before the vaccine became available and the concerns regarding a future unknown vaccine were high. Furthermore, once the COVID-19 vaccine became available, the effect of the pandemic on day-to-day life was remarkably high, and the authorities conducted a massive media campaign, officially named ‘Give a Shoulder’.

The main vaccination motivators in the survey were social and economic effects of the COVID-19 epidemic (‘I wish the morbidity rate will decrease and economic situation will recover’) as well as personal reasons (‘I am afraid to infect my family members with COVID-19’; ‘I am afraid to be infected by COVID-19’). The main concerns for hesitancy were vaccine safety and side effects, similar to the conclusions of Biswas et al.’s scope study that comprised 35 HCWs around the world and showed that more than 75% of them found concerns regarding safety, efficacy, and side effects [8]. These concerns resemble hesitancy in the general population, as shown in a Joshi et al. scoping review comprising 22 studies, 17 of which found safety and potential side effects of the COVID-19 vaccine to be a major concern [17].

Limitations of this study include a relatively small respondent group out of all the district employees. Furthermore, data regarding HCWs’ vaccination status did not include demographic variants and morbidity history due to ethical issues.

In summary, a high proportion of HCWs (88%) declared their positive attitude towards the COVID-19 vaccine, while the actual vaccination data were substantially lower (52%). These differences were high among the physicians (95% versus 63%); however, the largest differences were among the administration and logistics workers (87% versus 41%). Furthermore, the vaccination rate among HCWs was lower than the rate in the public during the same time period. The vaccination motivators were personal and social in similar percentages. Although these are partial data, to the best of our knowledge, this is the first study to compare survey data to true vaccination data.

We conclude that survey-based works should be interpreted cautiously, especially in HCW studies in which social desirability bias is enhanced, in which the prevailing opinion is pro-vaccination and expression of hesitancy is considered taboo. In reality of uncertainty, the concern about the unknown side effects and the new widespread vaccine technology carry weight in the decision to accept a new vaccine, even in the HCW community. We believe that increasing the advocacy efforts among HCWs, especially among nurses, such as conducting informative meetings and making information accessible, may increase the vaccination rates within the HCW population and may have a further positive effect on attitudes towards the vaccine among certain individuals in the general population.

## Data Availability

Except for statistical analysis, the data underlying this article cannot be shared publicly due to privacy issues such as the personal details of the study participants. The data will be shared on reasonable request to the corresponding author.

## Abbreviations

CHS: Clalit Health Services
CI: confidence interval
COVID-19: coronavirus disease 2019
EUA: Emergency Use Authorisation
FDA: The United States Food and Drug Administration
HCWs: healthcare workers
HMO: Health Maintenance Organization
OR: odds ratio
SARS-CoV-2: severe acute respiratory syndrome coronavirus 2
WHO: World Health Organization
